# A brief history of somatostatin interneuron taxonomy or: how many somatostatin subtypes are there, really?

**DOI:** 10.3389/fncir.2024.1436915

**Published:** 2024-07-17

**Authors:** Ariel Agmon, Alison L. Barth

**Affiliations:** ^1^Department of Neuroscience, Rockefeller Neuroscience Institute, West Virginia University, Morgantown, WV, United States; ^2^Department of Biological Sciences, Center for the Neural Basis of Cognition, Carnegie Mellon University, Pittsburgh, PA, United States

**Keywords:** somatostatin, cerebral cortex, inhibitory interneuron, transcriptomics (RNA sequencing), taxonomy

## Abstract

We provide a brief (and unabashedly biased) overview of the pre-transcriptomic history of somatostatin interneuron taxonomy, followed by a chronological summary of the large-scale, NIH-supported effort over the last ten years to generate a comprehensive, single-cell RNA-seq-based taxonomy of cortical neurons. Focusing on somatostatin interneurons, we present the perspective of experimental neuroscientists trying to incorporate the new classification schemes into their own research while struggling to keep up with the ever-increasing number of proposed cell types, which seems to double every two years. We suggest that for experimental analysis, the most useful taxonomic level is the subdivision of somatostatin interneurons into ten or so “supertypes,” which closely agrees with their more traditional classification by morphological, electrophysiological and neurochemical features. We argue that finer subdivisions (“t-types” or “clusters”), based on slight variations in gene expression profiles but lacking clear phenotypic differences, are less useful to researchers and may actually defeat the purpose of classifying neurons to begin with. We end by stressing the need for generating novel tools (mouse lines, viral vectors) for genetically targeting distinct supertypes for expression of fluorescent reporters, calcium sensors and excitatory or inhibitory opsins, allowing neuroscientists to chart the input and output synaptic connections of each proposed subtype, reveal the position they occupy in the cortical network and examine experimentally their roles in sensorimotor behaviors and cognitive brain functions.

## Introduction

Of the four subclasses of GABA-releasing inhibitory cortical interneurons, the somatostatin-containing group, colloquially referred to as “SOM cells” but more formally as SST interneurons (SST-INs), is the most diverse. For example, out of a total of 30 GABAergic “supertypes” identified by a recent large-scale transcriptomic study of mouse neocortical and hippocampal neurons, 12 are SST-INs (Yao et al., 2021a). However, SST-IN diversity was recognized well before the transcriptomic era. Earlier neuroanatomical studies referred to non-pyramidal cells in deep cortical layers with axons ascending to layer 1 as “Martinotti cells,” after their original 1889 description by Carlo Martinotti (e.g., Valverde, 1976; Szentagothai, 1978; Peters and Regidor, 1981). It was later discovered that Martinotti cells contained the neuropeptide somatostatin (Wahle, 1993). Using brain slice recordings, Kawaguchi and Kubota described diverse firing properties of two subtypes of somatostatin-containing Martinotti cells: “burst-firing non-pyramidal,” also called “low-threshold spiking” (LTS), and “regular-spiking non-pyramidal” (Kawaguchi and Kubota, 1996). Similar diversity of Martinotti firing patterns was reported by Wang et al. (2004), who also observed that some SST interneurons had axons which targeted layer 4 and not layer 1, but nevertheless retained the “Martinotti” eponym to refer to all SST-INs.

## GAD67-GFP transgenic lines: selective reporter expression in SST-INs

A turning point in recognizing SST-IN diversity came with the discovery of two SST-IN populations that did not extend axons to layer 1 and were therefore non-Martinotti cells. First, Tomioka et al. (2005) discovered a sparse population of nNOS-containing SST projection neurons, distributed in layers 2 and 6, that sent long-range axons to other cortical areas. Second, a study from our lab (Ma et al., 2006) described GFP-expressing cells in two novel GAD67-GFP transgenic lines we named X98 and X94, and compared them to a third GAD67-GFP line named GIN (Oliva et al., 2000). Although these lines were generated by random transgene integration, GFP expression in all three lines was restricted to SST-INs. Moreover, each line labeled a GFP-expressing subset with distinct laminar distribution, electrophysiological properties and neurochemical markers. Of these, the X94 subset was non-Martinotti, targeting layer 4 and not layer 1, and had a unique electrophysiological and neurochemical “fingerprint” that supported its consideration as a bona-fide, distinct SST subtype.

## SST-IRES-Cre driver line: ubiquitous reporter expression in SST-INs

The availability of mouse lines with GFP expression in specific SST subsets was a breakthrough, as it allowed different labs to repeatedly and consistently target the same population of identified neurons with a variety of experimental techniques, without the need for post-hoc verification of the identity of each neuron. In the years since their development, these three SST-GFP mouse lines were indeed used by multiple groups to examine morphology, physiology and development of SST-INs in *ex vivo* brain slices and *in vivo* (e.g., Halabisky et al., 2006; Fanselow et al., 2008; Hu et al., 2011; Gentet et al., 2012; Xu et al., 2013; Casale et al., 2015; Schmid et al., 2016; Donato et al., 2022). However, without the means to specifically express in these populations genetically encoded sensors or actuators, the roles of these distinct SST subsets in network or animal behavior could not be directly studied, with the notable exception of a study which used the elegant “Cre-DOG” system to express ChR2 in GFP-expressing X94 and GIN neurons (Naka et al., 2019). It is therefore not surprising that when a driver line expressing Cre in all SST interneurons was developed (Taniguchi et al., 2011) it was widely adopted by system neuroscientists. Over 560 published studies to-date (per Google Scholar) have used it, among other things, to “opto-tag” SST-INs *in vivo*, to record their calcium responses, or to activate/silence them optogenetically or chemogenetically. A handful of these studies found that SST-INs expressed a diversity of behavior-related activity profiles which corresponded to differences in their axonal distributions, their soma size, their laminar positions and/or their extracellular waveforms (Kvitsiani et al., 2013; Kim et al., 2016; Arriaga and Han, 2017; Muñoz et al., 2017; Yu et al., 2019), or that distinct SST-IN ensembles played opposing roles in behavior (Cummings et al., 2022), hinting at the underlying diversity within the SST population. In general, however, targeting all SST-INs *en masse* had the unintended consequence of hindering recognition of SST diversity.

## Transcriptomic taxonomies: an (over?)-abundance of subtypes

**FIGURE 1:**
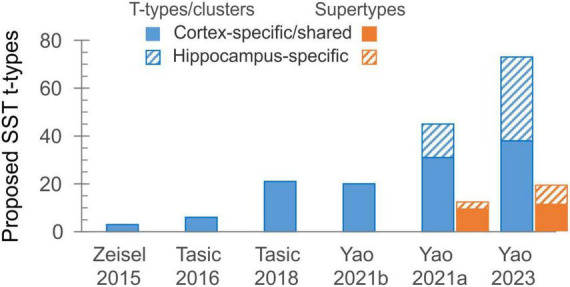
The exponential growth in the number of proposed transcriptomic types of SST interneurons over the last decade. The solid part of each bar represents types shared between isocortex and allocortex, or found wholly in isocortex.

While system neuroscientists were enthusiastically using a universal SST-Cre mouse driver line and (inadvertently) ignoring the diversity of SST subtypes, an increasing number of such subtypes were being discovered, thanks to newly developed single-cell and single-nucleus next-generation RNA sequencing technologies. These tools drove a large-scale effort, supported over the last ten years by the NIH through the BRAIN Initiative Cell Census Network (BICCN) (Ecker et al., 2017), to generate a comprehensive catalog and a high-resolution atlas of cell types in the human, primate and mouse brains, an effort which culminated recently with the whole-mouse-brain atlas.^1^ Along the way, researchers from the Allen Institute for Brain Science (AIBS) and other participating BICCN labs and institutions have been steadily expanding the number of cells sequenced, the cortical regions sampled, the sequencing depth and coverage, and the sequencing modalities used. As a result, the number of proposed transcriptomic types (“t-types” or “clusters”) of cortical SST (as well as other) interneurons has been rising exponentially, doubling or tripling from one study to the next (Figure 1), to the bewilderment and some consternation of researchers in the field. Starting with three types in one of the first large scale, single-cell RNA-Seq studies (Zeisel et al., 2015), the number jumped to six SST out of 23 GABAergic types (in visual cortex, Tasic et al., 2016), then to ∼20 SST out of ∼60 GABAergic types (in visual+anterolateral motor cortices, Tasic et al., 2018, or in primary motor cortex, Yao et al., 2021b), then in the very same year to 45 SST out of 123 GABAergic types (in cortex+hippocampus, Yao et al., 2021a). While 14 of the SST types in the latter study were restricted to hippocampus, the remaining 31 were either shared with or specific to isocortex. Recognizing the need for some simplification, the authors of the latter study introduced a new taxon below the “subclass” and above the “type” level, grouping the 45 SST t-types into 12 “supertypes.” In addition to the unique Chodl group (long-range projecting, nNOS-containing interneurons) and to two hippocampus-specific supertypes, these were named Etv1, Mme, Calb2, Myh8, Syndig1l, Hpse, Nmbr, Nts and Crh. Notably, while named after prominent marker genes they express, almost none of these genes show restricted expression to only one supertype (see Figure 1B in Wu et al., 2023).

## Martians and the African savannah: phenotypic vs. genotypic classification

We briefly digress to discuss the concept of “cell type.” In the era of transcriptome-based taxonomies, one almost forgets that long before these methods became available, neuroscientists classified neurons into types just like biologists, for the three centuries since Linnaeus (but indeed since the dawn of humanity), have classified organisms into species, genera, families etc., by their observable and measurable features—i.e., by their phenotypes. For neurons, these features include somatodendritic morphology (e.g., multipolar, bitufted, bipolar), axonal targets (e.g., soma-targeting, axon hillock-targeting or dendrite-targeting), intrinsic electrophysiological properties (e.g., input resistance, spike width, rebound spiking, repetitive firing patterns) and functional biochemical components such as neurotransmitters, neuropeptides, calcium-binding proteins, ion channels and receptors. Neuroscientists have settled on this constellation of properties as defining features of neuronal subtypes for two reasons. First, they can all be determined conjointly for any given neuron by recording from it electrophysiologically, filling it with dye, staining it with antibodies and examining it under light and electron microscopes. More fundamentally, these features describe how the neuron functions biophysically and biochemically, provide insight into its role in the wider network and allow researchers to predict how it would participate in, and contribute to, a given brain state or a specific animal behavior. In other words, characterizing neurons phenotypically is an important goal unto itself, not only a means for their classification. Knowing the transcriptomic signature of a neuron is not a substitute to its phenotypic characterization—at least, not until we learn how to predict the full complement of a neuron’s properties from its transcriptome, which despite recent headways (Cadwell et al., 2016; Tripathy et al., 2017; Nandi et al., 2022) remains a distant goal.

In this context, we can draw some illuminating parallels between the BRAIN Initiative-supported effort for a comprehensive transcriptomic taxonomy of cell types, and an even more ambitious “moonshot” effort, the Earth Biogenome Project (EBP), aiming to sequence and classify all eukaryotic species on earth.^2^ The EBP aims to generate “a complete Digital Library of Life that contains the collective biological intelligence of 3.5 billion years of evolutionary history” (Lewin et al., 2018). In addition to providing the annotated genome sequences for the 1.5 million or so known eukaryotic species, this initiative will ostensibly identify, sequence and classify all eukaryotic species yet unknown, a number that may easily exceed 10 million. But this extremely ambitious endeavor, still at its early stages, is not without its critics. Indeed, how much can one learn about a species, its anatomy, physiology and behavior, not to mention its interactions with other species within its ecosystem, from its genome alone? Would we be better off (as scientists, or as a society) if we sequenced fewer species, but used our inevitably limited resources to achieve a deeper understanding of their biology? As a *reductio ad absurdum*, imagine a parallel universe in which Martian scientists have developed a “Remote-seq” technology, and have compiled a massive MarsLab database of all earthling species, each with its complete genetic makeup. Unfortunately, despite this colossal effort over many Mars years, they still could not settle a long-standing controversy between Martian planetary zoologists about the food chain in the African savannah—does the leopard eat the gazelle, or does the gazelle eat the leopard?

Thankfully, AIBS scientists recognized the need to correlate transcriptomic taxonomies with observable neuronal properties, and embarked on a set of heroic “Patch-seq” experiments in which single-cell or single-nucleus RNA sequencing was applied to visual cortex neurons following *ex vivo* electrophysiological recordings and morphological reconstructions of the same cells (Gouwens et al., 2020). This resulted in a combined morpho-electric-transcriptomic (MET) classification which recognizes 13 SST MET-types, and it is reassuring that it maps reasonably well onto the 10 isocortical supertypes of Yao et al. (2021a) (Table 1, two leftmost columns).

**TABLE 1:** Correspondence between taxonomies.

Gouwens et al., 2020	Yao et al., 2021a	Yao et al., 2023	Genetic access
MET-1	Chodl	SST Chodl 4	
MET-2	Mme	SST 8	
MET-3, MET-4	Calb2	SST 10	Calb2-Cre; Sst-Flp
MET-5	Etv1	SST 11	
MET-6	Myh8	SST 4	Chrna2-Cre
MET-6	Syndig1l	SST 9	
MET-7, MET-8	Hpse	SST 7	X94
MET-9, MET-10	Crh	SST 1	Crh-Cre; Sst-Flp
MET-12	Nmbr	SST 5, 13, 16	Crhr2-Cre; Sst-Flp
MET-13	Nts	SST 14	

The most likely correspondence between MET-types (Gouwens et al., 2020) and supertypes (Yao et al., 2021a,^2023^); only supertypes derived wholly or mostly from isocortex are included. The correspondence with Yao et al. (2023) is based on a modified version of their Supplementary Table 7, provided by these authors. The last column lists mouse lines and intersectional strategies for accessing individual supertypes (based on Hostetler et al., 2023; Wu et al., 2023). Only strategies that appear to target single supertypes are included; for additional, less specific genetic strategies see Supplementary Table 3 of Wu et al. (2023).

## Flowers or leaves: the point of diminishing returns

In the ongoing push for ever-larger datasets and more comprehensive taxonomies, AIBS researchers have recently produced a whole-mouse-brain transcriptomic taxonomy and MERFISH atlas, each based on over 4 million cells, identifying about 5,300 neuronal and non-neuronal cell types organized in 1,200 supertypes (Yao et al., 2023). Of these, telencephalic GABA neurons occupy 1,050 clusters in nearly 300 supertypes. Examination of the “SST GABA” group (subclass #53) in this dataset, aided by the online Allen Brain Cell Atlas portal,^3^ reveals 73 SST clusters, 38 of which are exclusively or primarily from isocortex, arranged in 19 supertypes, 11 from isocortex (Figure 1; not including one isocortical Chodl supertype which is now placed in a different subclass). How the new supertypes relate to previous ones is shown in Table 1; however, the lack of descriptive labels to either clusters or supertypes (they are simply numbered 756–828 and SST GABA 1–19, respectively) limits the usability of this new taxonomy. Importantly, from the point of view of researchers in the lab, attempting to explain the variability in one’s experimental results by divvying the neuronal population under study into 73 (or even 38) distinct subtypes seems antithetical to why we may want to classify neurons to begin with—which is to reduce complexity in our data and impose order on the system under study. One might as well give up on classification altogether and assume that each of the studied cells is unique, as already concluded by the frustrated authors of an early study of hippocampal interneurons (Parra et al., 1998).

Should we keep splitting SST (and other) interneurons into ever more granular transcriptomic categories, when there are no reported functional distinctions between them? We may indeed have passed the point of diminishing returns. Notably, a large-scale Patch-seq study of motor cortex by another BICCN group has cast doubt on the notion that t-types are discrete entities altogether, and suggested that both gene expression profiles and morpho-electric features co-vary continuously within each subfamily (Scala et al., 2021). We concur, but suggest that the lowest meaningful taxon is the supertype. There is detailed structural and functional characterization for some—though not yet all–of the 10 isocortical supertypes (Ma et al., 2006; Hilscher et al., 2017; Nigro et al., 2018; Gouwens et al., 2020; Hostetler et al., 2023; Wu et al., 2023), and the correspondence between the 10 supertypes and the 13 MET types (Table 1) gives us hope that both schemes represent true groupings, reflecting robust, functionally important features unique to each group. Any finer subdivision, based on subtle differences in gene expression, may be artifacts of discrete sampling from a continuous distribution (Cembrowski and Menon, 2018; Tasic et al., 2018; Scala et al., 2021), and may reflect variability in the local environment such as cortical depth or rostrocaudal position (Yao et al., 2021a), in the brain state at the time the cells were harvested (Bugeon et al., 2022) or in their recent level of activity (Lacar et al., 2016; Wu et al., 2017; Hrvatin et al., 2018).

An analogy from plant taxonomy may be helpful here. At least since Linnaeus, botanists have recognized that the anatomical structure of the flower is a robust feature, showing great constancy within species, genera and families of plants. For example, nearly all members of the Cruciferae (mustard/cabbage) family have flowers with 4 sepals, 4 petals and 6 stamens. On the other hand, the shape of their leaves may vary widely between genera and between species within a genus. Even within a single species, leave shapes may vary with the local biotope (e.g., smaller in the sun and larger in the shade), or may change shape with the seasons, without changing their identity as a species. We believe that the recent taxonomies may have become overly concerned with leave shapes instead of flower structures.

## The true test of a subtype: its role in the network

Evolutionary logic suggests that just as biological species have evolved to fill ecological niches, neuronal subtypes must have evolved to perform distinct functions during sensorimotor behavior or cognitive processing (otherwise, there would be no need for them to diverge into different subtypes). To perform these functions, members of a given subtype must have evolved a distinct set of electrophysiological and morphological properties and also acquired a shared set of inputs and outputs, and as a result they should manifest a similar activity profile during a given behavior. Indeed, these predictions could be considered a stringent test of the validity of any neuronal classification system. To apply such tests, researchers need genetic access to putative subtypes—they need to tag them with fluorescent proteins, to monitor their electrical activity and to activate or silence them in the behaving animal. What is needed are high-fidelity driver lines (e.g., Cre, Flp, or Dre recombinase lines), or cell type-specific viral constructs, which accurately target reporter expression to distinct cell types. Unfortunately, such tools often lack the necessary sensitivity (they do not target all members of the group) or specificity (they also target members of other groups). Driver lines are typically generated by knocking-in the recombinase gene into an endogenous gene, thus recapitulating the expression pattern of that gene. Single genes, however, rarely define a cell type, and recapitulating the expression profile of a given gene will likely drive reporter expression in multiple cell types which may not even be closely related. For example, the Calb2-Cre line drives reporter expression in both SST and VIP interneurons, and the Calb1-Cre line labels both SST interneurons and upper layers excitatory cells. In our opinion, the lack of subtype-specific genetic tools is not only an impediment to proper neuronal classification, but may also be the biggest obstacle in the quest toward the ultimate goal of neuroscience—understanding the neural basis of perception, behavior and cognition.

## A path out of (or into) the jungle: intersectional genetics

To address the need for more specific targeting of interneurons in general and SST interneurons specifically, researchers have begun to use intersectional genetics. This approach involves crossing two (or more) driver lines with different recombinases (e.g., Cre and Flp) and injecting the dual recombinase progeny with a combinatorial reporter virus, or crossing them with a combinatorial reporter line. In some notable cases, this approach has successfully captured what appears to be *bona fide* cell types. Thus, the Sst-Flp; Calb2-Cre intersection limits reporter expression to Calb2-expressing SST cells, excluding the Calb2-expressing VIP interneurons. Neurons in the Sst-Flp;Calb2-Cre intersection have a distinct set of morphological and electrophysiological properties (He et al., 2016; Paul et al., 2017; Nigro et al., 2018; Hostetler et al., 2023; Wu et al., 2023) and seem to correspond to SST MET-3 and MET-4 types in layers 2/3 and 5, respectively (Gouwens et al., 2020), and to the Calb2 supertype (Yao et al., 2021a). A caveat, however, is that the Sst-Flp;Calb2-Cre intersection may label some Chodl neurons. Altogether, only a handful of transgenic lines and genetic intersections have so far been found to capture single, relatively “clean” transcriptomic SST supertypes (rightmost column in Table 1). New recombinase lines are needed, driven by judiciously selected genes, to provide more specific intersections; transcriptomic taxonomies themselves could be of immense value in identifying which marker genes to use in generating such driver lines. Even with new driver lines, we may find that the dual recombinase approach is simply not specific enough to capture a given subtype of interest, and while triple or even quadruple intersectional approaches are possible in principle, in practice the complicated breeding schemes required are likely to be too time-consuming and expensive for most labs. Some newly developed genetic tools, not relying on transgenic mouse lines, have shown promise in achieving cell-type-specific expression of payloads in mice, rats and primates, including enhancer-driven viral vectors (Dimidschstein et al., 2016; Hrvatin et al., 2019; Vormstein-Schneider et al., 2020; Graybuck et al., 2021; Mich et al., 2021), and the unique CellREADR system which directly detects cell-type-specific mRNAs (Qian et al., 2022). These and additional, as yet undeveloped methods are needed, if we are to assemble a toolbox that will allow genetic access to all supertypes of SST (and other) interneurons.

## Concluding remarks

The very first of the stated goals of the 2014 NIH-sponsored BRAIN Initiative was to “**Identify and provide experimental access** to the different brain cell types to determine their roles in health and disease” (bold lettering added by us).^4^ Much emphasis over the last ten years has been on cell-type identification, and we suggest that a shift to providing experimental access to identified cell types is needed as we progress to BRAIN 2.0.^5^ Providing experimental access to the major supertypes is within our reach, and well-worth the required effort and resources. Achieving this goal will open up Cajal’s “impenetrable forest” to a new generation of intrepid explorers who will go beyond cataloging its biodiversity, to unraveling the intricate web of life that binds its myriads of species into one holistic ecosystem.

## Data availability statement

The original contributions presented in this study are included in this article/supplementary material, further inquiries can be directed to the corresponding author.

## Author contributions

AA: Writing – original draft, Writing – review & editing. ALB: Writing – review & editing.

## Footnotes

1
https://www.biccn.org/science/whole-mouse-brain

2
https://www.earthbiogenome.org

3
https://knowledge.brain-map.org/abcatlas

4
https://braininitiative.nih.gov/vision/nih-brain-initiative-reports/brain-2025-scientific-vision

5
https://braininitiative.nih.gov/vision/nih-brain-initiative-reports/brain-20-report-cells-circuits-toward-cures#Heading7
